# Medical and non-medical reasons for cesarean section delivery in Egypt: a hospital-based retrospective study

**DOI:** 10.1186/s12884-019-2558-2

**Published:** 2019-11-08

**Authors:** Shatha Elnakib, Nahla Abdel-Tawab, Doaa Orbay, Nevine Hassanein

**Affiliations:** 1 Department of International Health, Johns Hopkins Bloomberg School of Public Health, 615 N. Wolfe Street, Baltimore, MD 21205 USA; 2 Population Council, Cairo, Egypt; 3 Independent Consultant, Reproductive Health Consultant, Cairo, Egypt

**Keywords:** Egypt, Maternal health, Reproductive health, Caesarean section, Indications

## Abstract

**Background:**

Caesarean section (CS) is an important lifesaving intervention that can reduce maternal and newborn morbidity and mortality. The dramatic increase in CS rates globally has prompted concerns that the procedure may be overused or used for inappropriate indications. In Egypt, CS rates are alarmingly high, accounting for 52% of all deliveries. This study sought to (1) explore indications and risk factors for CS in public hospitals in four governorates in Egypt and (2) examine health care provider factors impacting the decision to perform a CS.

**Methods:**

We reviewed medical records for all deliveries that took place during April 2016 in 13 public hospitals situated in four governorates in Egypt (Cairo, Alexandria, Assiut and Behera), and extracted information pertaining to medical indications and women’s obstetric characteristics. We also interviewed obstetricians in the study hospitals to explore factors associated with the decision to perform CS.

**Results:**

A total of 4357 deliveries took place in the study hospitals during that period. The most common medical indications were previous CS (50%), an “other” category (13%), and fetal distress (9%). Multilevel analysis revealed that several obstetric risk factors were associated with increased odds of CS mode of delivery – including previous CS, older maternal age, and nulliparity – while factors such as partograph completion and oxytocin use were associated with reduced odds of CS. Interviews with obstetricians highlighted non-medical factors implicated in the high CS rates, including a convenience incentive, lack of supervision and training in public hospitals, as well as absence of or lack of familiarity with clinical guidelines.

**Conclusion:**

A combination of both medical and non-medical factors drives the increase in CS rates. Our analysis however suggests that a substantial number of CS deliveries took place in the absence of strong medical justification. Health care provider factors seem to be powerful factors influencing CS rates in the study hospitals.

## Background

Caesarean section (CS) is an important lifesaving operation for both mother and child, and its use has increased dramatically over the last decade [1]. Mirroring global trends, CS rates in Egypt have steadily increased, reaching 52% of all deliveries according to the most recent 2014 Egypt Demographic and Health Survey (EDHS) and representing more than a 100% increase in the CS rate since 2005 [2]. The proportion of institutional-based CS is 67.3%, which is more than double that of Jordan and Saudi Arabia, Egypt’s regional neighbors [3]. Currently, Egypt has the third highest rates of CS globally, following the Dominican Republic (56.4%) and Brazil (55.6%) [1].

According to the Statement on Caesarean Section Rates released by the World Health Organization, population-based CS rates greater than 10% are not optimal [4]. Although WHO has indicated that countries should not strive to achieve a specific rate, the rationale for the 10% recommendation is based on a systematic review and ecological analysis which have shown that CS rates exceeding 10% are not correlated with reductions in maternal and newborn mortality [5, 6]. Instead high CS rates may increase maternal risks, adversely impact future pregnancies and overstretch health systems [7, 8]. According to a 2010 report, the global cost of excess CS is US$ 2.32 billion [9].

Reasons behind the global increase in CS are multifaceted and include both clinical and non-clinical factors. Changes in risk profiles of women, a purported rise in medical indications as well as non-medical reasons including social, cultural and economic factors underlie the increase in CS rates in many settings [10–13]. Another factor implicated in the increase in CS rates is the “physician factor,” which attributes the rise in CS not to obstetric risk factors, but to physician-related and institutional reasons [14–16].

In Egypt, significant progress has been achieved with regards to maternal health and safe motherhood. The maternal mortality rate declined from 106 per 100,000 live births in 1990 to 45 per 100,000 in 2013 [17]. According to EDHS 2014, coverage of antenatal care increased to 90% and facility delivery increased to 87% - a marked improvement compared to EDHS 2008 in which 71.6% of deliveries took place in a health facility [18, 19]. It is worth noting that the last round of EDHS revealed that most deliveries were assisted by doctors (88%) and only 3% were assisted by nurse-midwives [19]. As such, the role of nurse-midwives in delivery is limited. Hospital nurses typically have a very narrow scope of work and their role in the delivery room is restricted to assisting the obstetrician. Nurse/midwives, on the other hand, receive midwifery training and are certified to conduct childbirth at home or at the primary health care facility to which they are affiliated [20, 21]. Cases however are limited to those that are low-risk, while high-risk cases are referred to the nearest hospital.

In 2016 – the year in which this study was carried out – a total of 2,600,173 deliveries took place in Egypt, most of which occurred in health facilities [22]. Concurrent with the increase in facility-based deliveries, CS rates have increased at an alarming rate [3]. The high rate of CS delivery is concerning in light of the association of non-medically indicated CS with maternal and fetal complications [23, 24] and suggests that caesarean delivery might be overused or used for inappropriate indications. Key to ensuring that CS is being performed for appropriate clinical reasons is routine monitoring and audits of medical record data from institutional deliveries. Population studies rarely provide accurate information on indications for CS delivery nor on the obstetric characteristics of women undergoing CS [25–27]. Indeed, there is a general paucity of studies elucidating physician-documented indications [28, 29] and in Egypt, this is coupled with a lack of data illustrating the obstetric risk factors for CS delivery. The most recent study analyzing CS trends in Egypt noted that the exponential increase in CS deliveries necessitates an institutional-based examination of the medical and non-medical drivers of CS to allow for a deeper understanding of why the CS rate is increasing and what can be done to curb this increase [3]. To understand medical indications and health care provider factors associated with the high CS rates, we conducted a retrospective review of medical records of all deliveries that took place in 13 public hospitals situated in four governorates in Egypt. The review was triangulated with structured interviews with 275 physicians in the study hospitals. The specific aims of the study were two-fold: [1] to explore documented indications and risk factors for CS in a sample of public hospitals and [2] to examine health care provider factors that may be responsible for the increased CS rates, such as financial and non-financial incentives, training, and supervision.

## Methods

### Study setting

**Fig. 1:**
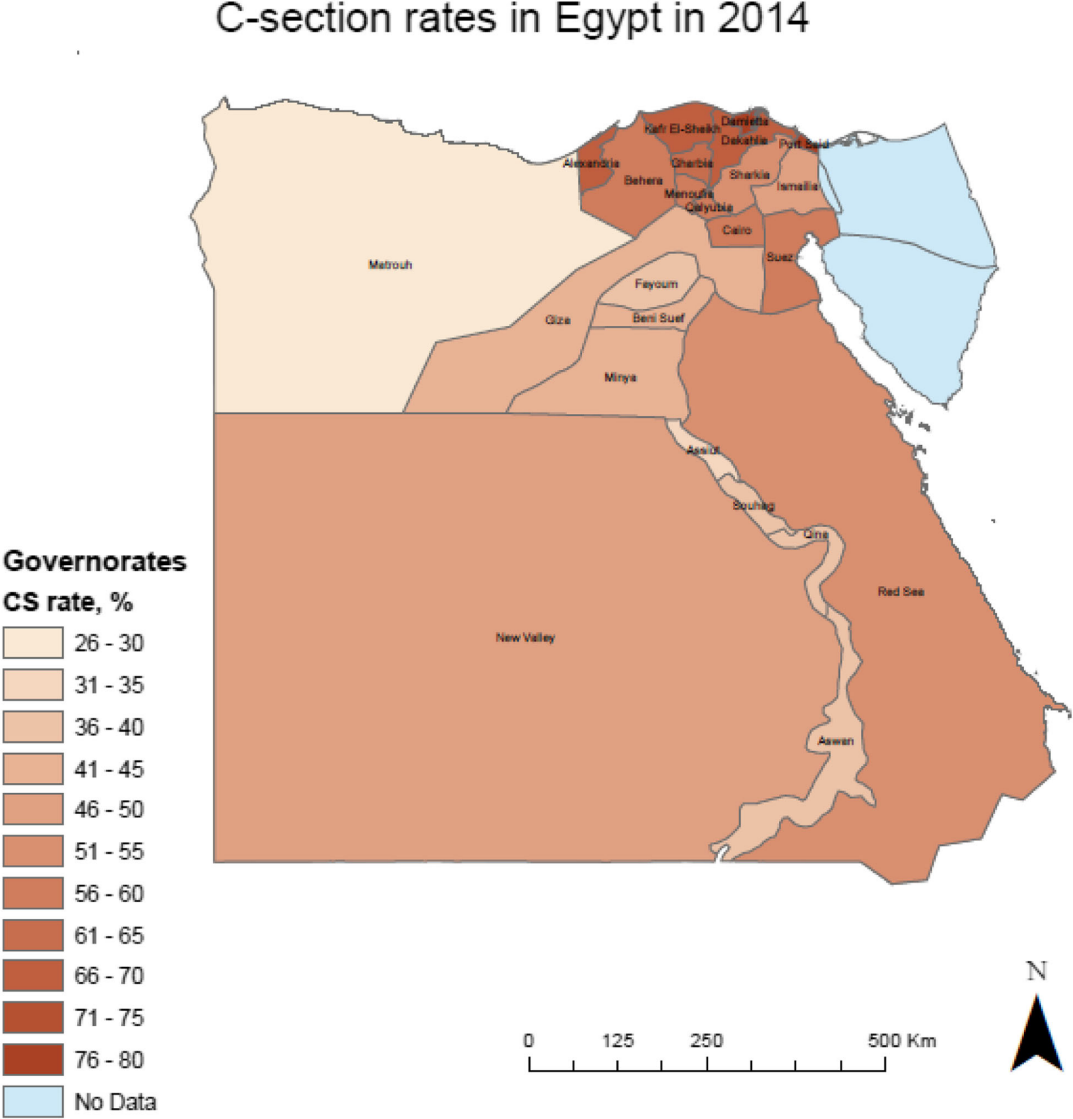
C-section rates in Egypt by governorate according to EDHS 2014. We generated the map of Egypt using ArcMap 10.6 to depict the distribution of CS rates by governorate

A total of 13 public hospitals were selected in four governorates – one in Upper Egypt, one in Lower Egypt, and two Urban Governorates. The study governorates were Assiut (*n* = 3 hospitals), Behera (*n* = 3 hospitals), Alexandria (*n* = 3 hospitals), and Cairo (*n* = 4 hospitals). CS rates vary across the four governorates, ranging from 35 to 68% according to the most recent 2014 EDHS data (See Fig. 1).

Medical records were abstracted for all deliveries that took place in the study hospitals during the month of April, 2016.

Both the study protocol and data collection instruments were reviewed and approved by the Population Council’s Institutional Review Board and the Ethics Committee of the Egyptian Ministry of Health and Population (MoHP). IRB approval was obtained on February 2nd, 2016 (protocol #722).

### Study sample

Data collection in the 13 hospitals over the month of April 2016 yielded a total of 4357 medical deliveries. Additionally, structured interviews with all obstetricians who were available during the data collection period and who worked in the Ob/Gyn ward of the study hospitals – were conducted. A total of 275 physicians were interviewed to identify physician and hospital factors related to the performance of CS in the study hospitals.

### Data collection

Data from medical records – which are paper-based in Egypt – were collected using an abstraction form. Junior residents were trained in the respective hospitals on extracting data from hospital archives and filling the abstraction forms provided. They were instructed to get back to physicians and nurses if they encountered missing data. Population Council staff conducted regular monitoring visits to ensure correct data extraction. Data from medical records was recorded anonymously. The abstraction form included patient profile variables, history of mode of delivery – specifically history of previous CS, as well as maternal and fetal medical indications. Data collectors recorded the indication exactly as it was listed in the patient file. ICD-10 codes for medical indications were grouped into the following categories [8]: 1. Fetal distress/non-reassuring fetal status, 2. abnormal lie, 3. amniotic fluid disorder including oligo and poly-hydramnios, 4. macrosomia, 5. multiple gestation, 6. prolonged and obstructed labor including cervical dystocia, 7. previous CS, 8. hypertensive disorders including eclampsia, preeclampsia and hypertension, 9. maternal disorders including hear problems and liver disease, 10. antepartum hemorrhage including placenta previa, 11. infection and fever and, 12. an “other” category.

Structured face-to-face interviews with obstetricians were administered by a trained researcher. A questionnaire comprising 40 close-ended questions was used to guide the interview. Specifically, obstetricians were asked about the preferred mode of delivery in their hospital, decision-making processes, and perceived changes in mode of delivery over time. They were also asked about hospital level factors that may influence CS such as the presence of incentives favoring CS deliveries – both material and others, availability of medical protocols and training, and the conduct of medical reviews or audits.

### Statistical analysis

Obstetric risk factors highlighted in the literature and for which data was available in medical records were analyzed using univariable and multivariable multilevel logistic modelling. Two separate outcomes were assessed: pre-labor CS and CS after onset of labor. The dataset was hierarchical with 2 levels, patients as the first level and hospitals as the second level. The use of a random intercept in a logistic regression model allowed us to cluster the effects of the patient’s characteristics on the probability of undergoing a CS across hospitals. Prevalence odds ratios (OR) with a 95% confidence interval (95% CI) were obtained. The model is specified as follows (Eq. 1).

1 $$ {\mathrm{Y}}_{\mathrm{ij}}=\upbeta 0+\mathrm{b}0\mathrm{i}+\upbeta 1\mathrm{x}1\mathrm{ij}+\upbeta 2\mathrm{x}2\mathrm{ij}+\cdots +\upbeta \mathrm{nxnij}+\upvarepsilon 0\mathrm{i} $$

Where Y is the probability of CS for individual j and hospital I; X are predictor variables, β is the estimated coefficient corresponding to X, and b0i is the random intercept.

The intraclass correlation coefficient (ICC) was also calculated (Eq. 2). This measure expresses the fraction of variance of the dependent variable that is due to differences between hospitals.

2 $$ \mathrm{ICC}=\frac{\sigma 2}{\sigma 2+\frac{\pi 2}{3}} $$

## Results

### Medical records

**Fig. 2:**
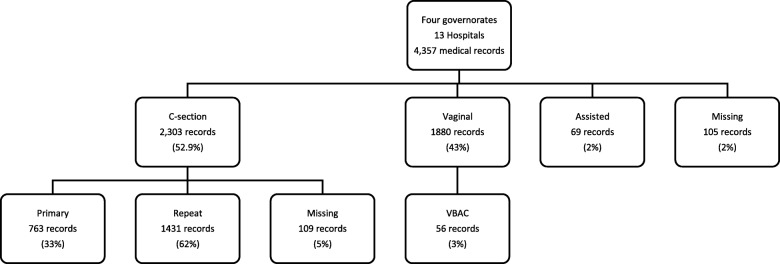
Distribution of medical records across the 13 hospitals

A total of 4357 records of women who gave birth during the month of April 2016 were obtained in the 13 public hospitals in Assiut, Behera, Alexandria and Cairo. Around 2% of medical records had missing data on mode of delivery. Of the 4252 records for which a mode of delivery was recorded, CS delivery accounted for more than half of deliveries (54.2%) (Fig. 2). Almost 10% of medical records for CS deliveries had no medical indications listed. Around 49% of CS deliveries occurred before onset of labor.

**Fig. 3:**
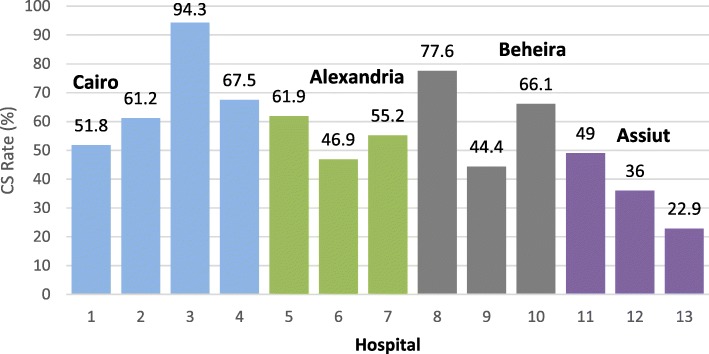
CS rates in each of the study hospitals in April 2016

A significant heterogeneity was observed in CS rates across the 13 hospitals with hospital-specific rates ranging from a low of 22.9% to a high of 94.3% (Fig. 3).

**Fig. 4:**
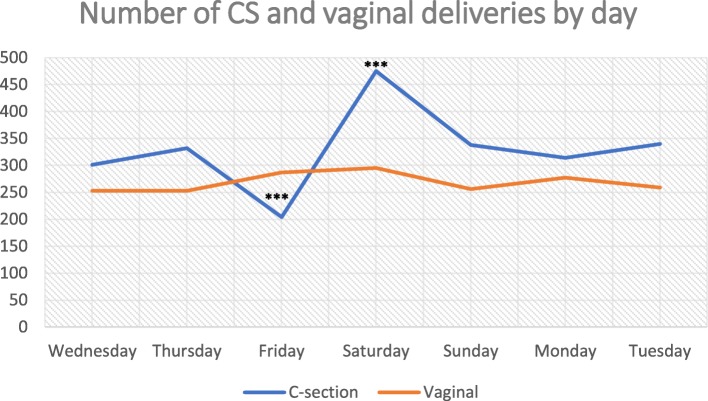
Number of CS deliveries performed by day of the week. ***Denotes *p*-value< 0.001 with regards to comparison with the day before

Fig. 4 shows number of caesarean and vaginal deliveries in the study hospitals by day of the week. The plot reveals that CS deliveries peaked on Saturdays (considered the first working day in the public sector) and were lowest on Fridays (the first day of the weekend and considered a holy day), whereas for vaginal deliveries, there were no marked differences based on day of the week.

### Medical indications

**Fig. 5:**
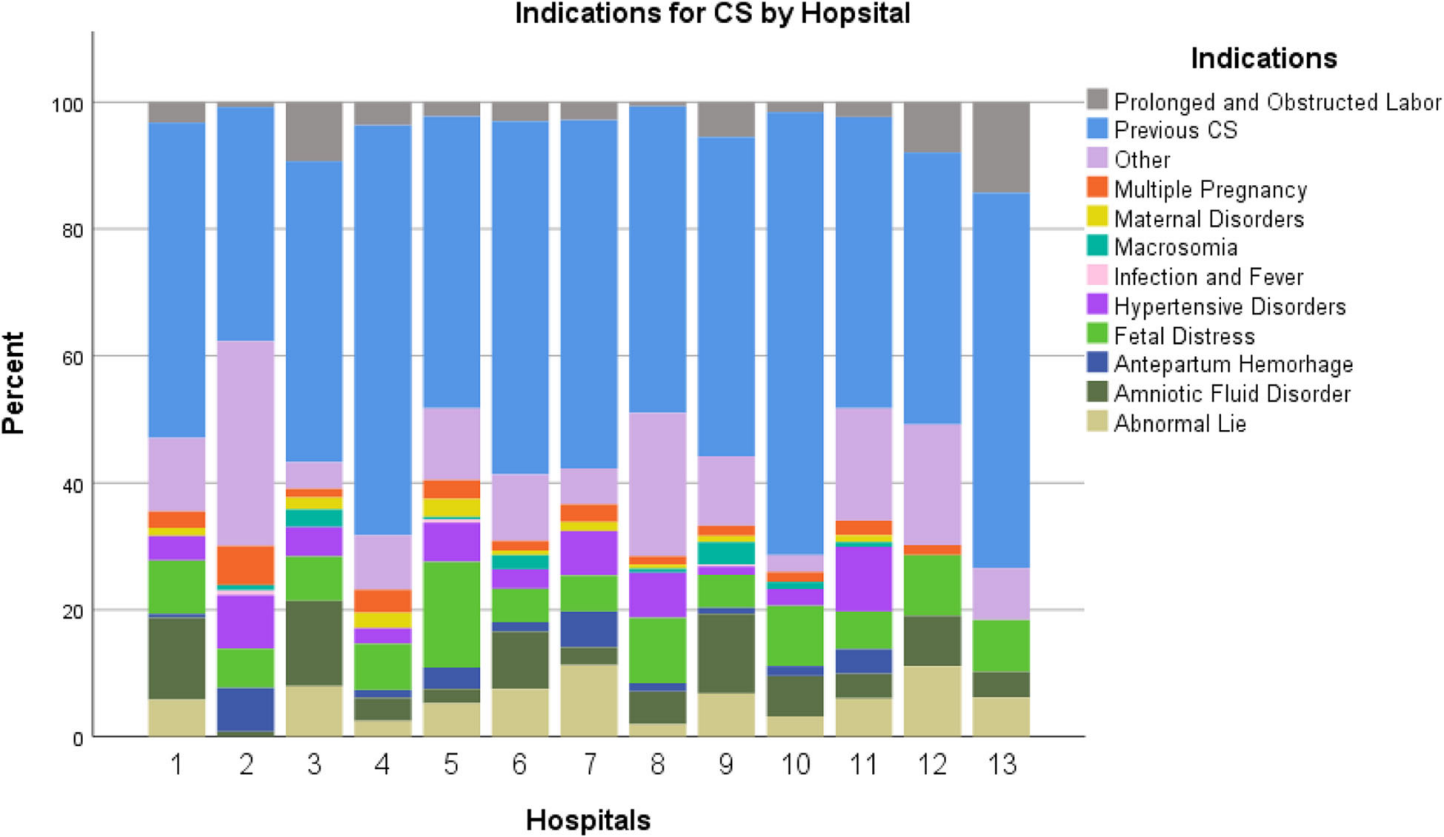
Distribution of medical Indications for CS by study hospital

Fig. 5 demonstrates the distribution of maternal and fetal indications for CS according to hospital records. Almost half of all recorded medical indications were previous CS (50%), followed by an “other” category (13%), and fetal distress (9%). Notably, out of a total of 1259 previous CS indications, 1047 did not have any other accompanying indications listed. For the most part, there were no remarkable differences in the distribution of medical indications across the study hospitals.

Overall partograph use was very low in the study hospitals. Availability and completion of a partograph was checked for all medical records. Only 397 of records (9.11%) contained a completed partograph, of which 216 were for CS deliveries and 127 were for vaginal deliveries. Among pre-labor CS deliveries, a partograph was available for 14.9% of the cases.

### Obstetric profile of women undergoing CS in the study hospitals

The obstetric characteristics of women delivering by CS vs those delivering vaginally are shown in Table 1.

**Table 1:** Characteristics of women undergoing Caesarean section delivery vs vaginal delivery in the study hospitals

Characteristic	*N* = 4183	
	Vaginal Delivery *n* = 1880	Caesarean Delivery *n* = 2303
Maternal Age at delivery		
Less than 21	357 (19.2%)	249 (11.4%)
21–34	1321 (71%)	1622 (74%)
35 and above	184 (9.9%)	322 (14.7%)
Gestational Age		
< 37 weeks	188 (10.8%)	365 (16.9%)
≥ 37 weeks	1559 (89.2%)	1798 (83.1%)
Parity		
Nulliparous	502 (29.4%)	456 (21%)
Multiparous	1206 (70.6%)	1721 (79%)
Previous CS		
Yes	56 (3%)	1431 (65.2%)
No	1787 (97%)	763 (34.8%)
Multiple Gestation		
Singleton	1766 (95.1%)	2035 (91.9%)
Multiple	92 (5%)	180 (8.1%)
Eclampsia		
Yes	10 (0.53%)	101 (4.4%)
No	1870 (99.5%)	2202 (96%)
Fetal Presentation		
Cephalic	1773 (95.6%)	1852 (86.5%)
Non-cephalic	82 (4.4%)	290 (13.5%)
Onset of Labor		
Yes	1852 (100)	993 (51%)
No	0 (0%)	954 (49%)

The intraclass correlation due to hospital accounted for 26% of the variance in pre-labor CS, and 19% of the variance in CS with labor onset. Table 2 presents results of the risk factor analysis of CS with and without onset of labor.

**Table 2:** Obstetric risk factors associated with Caesarean section with and without onset of labor compared with vaginal delivery

Risk Factor	CS with onset of labor		CS without onset of labor (pre-labor)	
	OR (95% CI)	AOR (95%CI)	OR (95% CI)	AOR (95%CI)
Maternal age at delivery				
Less than 21	Ref	Ref	Ref	Ref
21–34	1.5 (1.2–1.9)	1.7 (1.1–2.5)	1.7 (1.3–2.1)	1.63 (1.1–2.3)
35 and above	1.8 (1.3–2.5)	2.1 (1.3–4.0)	2.7 (2.0–3.7)	3.9 (2.3–6.5)
Gestational age				
< 37 weeks	Ref	Ref	Ref	Ref
≥ 37 weeks	0.9 (0.7–1.1)	1.9 (1.3–2.9)	0.6 (0.5–0.8)	1.05 (0.71–1.56)
Parity and previous CS				
Nulliparous	Ref	Ref	Ref	Ref
Multiparous with previous CS	30.6 (21.7–43.2)	13.3 (8.4–20.9)	32.7 (23.0–46.4)	36.5 (24.2–55.2)
Multiparous without previous CS	0.3 (0.2–0.4)	0.2 (0.1–0.3)	0.2 (0.2–0.3)	0.2 (0.1–0.2)
Multiple Gestation				
Singleton	0.8 (0.6–1.2)	2.1 (1.0–4.4)	0.8 (0.6–1.2)	2.1 (1.1–4.15)
Multiple	Ref	Ref	Ref	Ref
Fetal Presentation				
Cephalic	Ref	Ref	Ref	Ref
Other	4.0 (2.7–5.8)	8.5 (4.7–15.5)	3.2 (2.3–4.6)	10.7 (6.1–18.7)
Eclampsia				
Yes	4.0 (1.9–8.6)	5.5 (1.9–15.4)	11.9 (6.0–23.7)	24.1 (10.1–57.6)
No	Ref	Ref	Ref	Ref
Oxytocin use				
Yes	0.4 (0.2–0.7)	0.1 (0.06–0.12)	–	–
No	Ref	Ref		
Partograph				
Yes	0.4 (0.2–0.8)	0.3 (0.1–0.8)	–	–
No	Ref	Ref		

*OR* odds ratio; *AOR* adjusted odds ratio

Maternal age at delivery was a strong risk factor for CS among women who delivered by CS with onset of labor; for a given hospital, women ages 35 and older had 2.1 times the odds of CS delivery compared to women younger than 21 (95% Confidence Interval [CI] 1.3–4.0), and women 21–34 years had 1.7 times the odds of CS (95% CI 1.3–2.1) compared to women below age 21. Similarly, there was a strong association between age and pre-labor CS delivery. This association was strongest for women ages 35 and older (Adjusted OR [AOR] 3.9).

Among women who underwent labor, high gestational age (≥37 weeks) was positively associated with CS (AOR 1.9, 95% CI 1.3–2.9). This association was not present for women who did not experience labor (AOR 1.05, 95% CI 0.71–1.56). The strongest risk factor for CS delivery was previous CS among multiparous women. This association was strong for women who underwent labor (AOR 13.3) and even stronger among women whose CS occurred before onset of labor (AOR 36.5). Interestingly, women who were multiparous and had no history of previous CS had significantly lower odds of delivering by CS, compared to their nulliparous counterparts. Moreover, singleton births were associated with decreased odds of CS compared to multiple births in unadjusted analysis (OR 0.8); nonetheless, after adjustment, the odds ratio of CS delivery among women who delivered a singleton birth was 2.1 (95% CI 1.0–4.4) in the presence of labor and 2.1 (95% CI 1.1–4.15) in the absence of labor. The confidence interval, however, overlaps the null value for the former.

Non-cephalic fetal presentation and eclampsia were both strongly associated with CS mode of delivery. These associations however were stronger for pre-labor CS deliveries. Further, oxytocin which is used to induce and augment labor was negatively associated with pre-labor CS (AOR 0.1, 95% CI 0.06–0.12), and so was the use of a partograph (AOR 0.3, 95% CI 0.1–0.8).

OR, odds ratio; AOR, adjusted odds ratio.

### Structured interviews with physicians in the study hospitals

A total of 275 obstetricians in the study hospitals were interviewed. Their characteristics are presented in Table 3.

**Table 3:** Demographic characteristics of the sample (*N* = 275)

Characteristics	Obstetricians
Title, n (%)	
Resident	116 (42.34)
Assistant Specialist	56 (20.44)
Specialist	63 (22.99)
Consultant/Professor	39 (14.23)
Sex, n (%)	
Male	165 (61,57)
Females	103 (38.43)
Age, n (%)	
20–29	61 (22.5)
30–39	111 (40.96)
40–49	45 (16.61)
50–59	41 (15.13)
60+	13 (4.80)
Years of Experience, mean ± sd	11.16 ± 10.26

Around 88% of respondents confirmed that they observed an increase in CS rates in their respective hospitals and 60% confirmed that some cases that are delivered by CS could have been delivered vaginally. Providers mainly attributed the increase in CS to a rise in cases with medical indications (71%) followed by maternal request for elective caesarean (42%), physicians’ personal preference (21%) and lastly reasons related to hospital systems and resources (10%).^1^ When asked about indications most commonly responsible for CS delivery, 91% answered “previous CS.” Around 25% listed fetal status as a common medical indication, followed by fetal lie (18%), maternal factors (18%), placental reasons (12%), and failure to progress (12%).^2^

When asked specifically if obstetricians in their hospital preferred a CS to vaginal delivery, 42% of respondents answered affirmatively. The main reasons for preferring a CS were doctors’ ability to schedule the CS at their convenience (44%), the shorter duration of delivery by CS compared to vaginal delivery (28%), inadequate training of physicians in vaginal delivery (44%) and financial incentives (17%).

Asked whether there are standard guidelines for mode of delivery in their respective hospitals, 39% stated that there were none and 4% answered that they did not know of any.

In terms of training in CS, when asked if residents are expected to perform a minimum number of supervised CS deliveries before operating independently, only 45% of participants indicated that their hospitals specify a minimum number of CS cases that a resident must perform under supervision before s/he is allowed to work on his/her own. With respect to morning rounds, review of management of high-risk cases or staff meetings in which residents can discuss deliveries with more senior staff, 25% of physicians stated there weren’t any such opportunities and 7% stated that these were not regular. Additionally, 23% of respondents indicated that their hospitals did not receive medical audits or supervisory visits from higher level agencies or authorities.

## Discussion

In this study, the average CS rate was 53%, but varied across hospitals ranging between 22.9 and 94%. Some variation in CS rates and in the distribution of medical indications should be reasonably expected considering inherent differences in the patient population case-mix at each hospital as well as the size and nature of the hospitals (e.g. district versus teaching or university hospital^3^).

Inspection of medical indications indicated that CS was being performed in the absence of strong medical justification. Across study hospitals, the most commonly cited indications were previous CS, ‘other’, and fetal distress. Two of these should not result in CS by default, namely previous CS – for which a trial of labor after caesarean is a well-recognized option – and fetal distress, for which there are several interventions that can be attempted before a decision is made to opt for CS [8, 30]. We found very high rates of repeat CS which could be behind the alarming increase in overall CS rates. According to the American College of Obstetricians and Gynecologists, previous CS should not be an indication in the absence of any obstetric emergencies [31]. Yet, previous CS was the leading indication for CS in all study hospitals, and around 1047 CS deliveries only had “previous CS” listed as an indication. Additionally, the majority of interviewed obstetricians confirmed that previous CS was a common indication motivating the choice of delivery. A multitude of studies have shown that vaginal birth after caesarean (VBAC) is a safe option, with good success rates and low associated risk [32–34]. In several countries, a trial of labor after one CS is recommended in an effort to curb the increase in CS rates [31, 35]. In this study, the VBAC rate constituted a meager 3% of deliveries with previous CS. It is therefore important that physicians are encouraged to implement evidence-based obstetric practices, such as VBAC, instead of automatically opting for CS.

Additionally, more subjective indications, such as labor abnormalities without a completed partograph and non-reassuring fetal status or fetal distress without a documented fetal heart rate tracing or monitoring were commonly recorded as indications. In light of the low rates of partograph use and low fetal cardiac monitoring in the study hospitals, indications such as non-reassuring fetal status for example seem to be largely based on subjective clinician’s judgement instead of a completed partograph. These findings seem to contrast with what physicians had reported when asked about perceived reasons behind the increase in CS in their hospitals. According to them, the primary reason for the rise in CS was the frequency of medical indications requiring a CS. However, the lack of objective medical indications which unequivocally warrant this mode of delivery casts some doubt on that view.

With respect to investigations informing choice of mode of delivery, a partograph, which is designed to help obstetricians make the right decision for intervention in the right time was only done for 9% of all deliveries and 14.9% of CS deliveries in which labor was experienced. Lack of a partograph may indicate that there was little effort being made to document, monitor and assess progress of labor and other fetal conditions, and that instead physicians were opting for CS by default. In light of evidence that a partograph can help in reducing CS rates [8, 36], especially among women experiencing delays in the progress of labor [37], a partograph may be a valuable tool in this context.

Findings from our multilevel regression analysis are consistent with previous research on risk factors for CS and shed light on obstetric factors associated with CS delivery in this context. Regression analysis revealed that obstetric history strongly influenced mode of delivery. Similar to other studies, previous CS and nulliparity were strongly associated with CS both among women who experienced labor and those who did not [38, 39]. Nulliparity was found to be strongly associated with CS delivery, which is worrisome because it indicates that women with no history of childbirth are increasingly delivering by CS. Additionally, older maternal age was a risk factor for CS delivery. Even though childbearing begins early for many Egyptian women, age at first birth is increasing which may raise concerns about further expansion in the CS rates [19]. Not surprisingly, obstetric risk factors such as non-cephalic presentation and eclampsia were associated with increased odds of CS delivery. Gestational age was only associated with pre-labor CS, but was not a risk factor for CS without onset of labor. In line with other studies, our analysis further confirms that partograph use and oxytocin are associated with decreased odds of CS [40–42].

In addition to medical and obstetric risk factors identified from the analysis of medical records, interviews with obstetricians shed light on several provider-related drivers of CS. The analysis revealed three important factors that could be contributing to the expansion in CS rates: [1] a convenience incentive; [2] lack of supervision and training; [3] and absence of or lack of familiarity with clinical guidelines. A little less than half of obstetricians confirmed a personal preference for CS. Interviewed physicians cited the shorter duration of a CS compared to a vaginal delivery as a reason for why CS might be favored. This combined with the ability of doctors to decide on the timing of the delivery make CS a more convenient option for physicians. That most CS deliveries in this study took place on a Saturday, which is the first working day of the week where most staff are available, and rarely on Friday – which is considered a holy day and a day of rest, is evidence that physicians may prefer CS owing to scheduling preferences. In comparison, vaginal deliveries were equally likely to occur at any given day of the week.

Our study also pointed to the lack of training and performance supervision in the hospitals, both within the hospital through obstetric peer review as well as from outside the hospital through medical audits and supervisory visits from higher authorities. Regular supervision, in the shape of staff rounds and meetings, can offer an opportunity for the review of deliveries and enable the provision of individual feedback to providers. Staff rounds can also serve as a learning exercise through which younger obstetricians can learn from the experiences of others. Such mechanisms should be used to institutionalize a system of routine monitoring of physicians’ practices while serving as an accountability mechanism to hold those who perform unjustified CS accountable. By requiring a mandatory second opinion, hospitals may also be able to control the CS rates without causing adverse effects on maternal and neonatal outcomes. Both obstetric peer review strategies as well as mandatory second opinion have been shown to reduce CS rates in other settings [43, 44]. Some providers also viewed poor training in vaginal delivery as a reason behind the tendency to resort to CS. This finding aligns with other literature which indicates that loss of medical competence in attending a vaginal delivery is a driver of increasing CS rates [45].

Moreover, the finding that nearly 43% of physicians are either not aware of the presence of standardized guidelines in their respective hospitals or claim they do not exist to begin with attests to the urgent need for dissemination of standardized clinical guidelines and for their activation in hospitals. Unfortunately, available national guidelines developed by the MoHP for Ob/Gyns are general and cover a broad set of topics including antenatal care, delivery, post-natal care, pre-eclampsia, among others. Further, they were last updated almost 10 years ago. Hence, the need for updated national guidelines and evidence-based recommendations is pressing, especially with respect to antenatal and intrapartum management of VBAC deliveries. Raising the awareness of providers about the appropriate indications for CS and the importance of advocating for vaginal delivery among eligible women – including those with a previous CS – can trigger practice changes that may reduce the incidence of non-medically indicated caesarean delivery.

Finally, maternal request was cited by 42% of physicians as a reason for the increase in CS rates. Unfortunately, maternal request is not readily identifiable in medical records and women were not asked about this in our study. Hence, we were not able to verify this statement.

## Limitations

Study hospitals were not randomly selected and are not nationally representative, which limits the generalizability of our findings. Additionally, the use of medical indications has its limitations. For one, there may be a lack of uniformity in the definition of medical indications and variability in record keeping across study hospitals. Also, false reporting of complications and exaggeration of medical indications to justify a CS procedure are possible. Our analysis still reveals that the most common medical indications, particularly previous CS, do not lend strong medical justification for CS delivery, and that commonly recorded medical indications were more likely to be subjectively defined and based on a clinician’s judgement.

Another limitation is that medical records lacked information on maternal request, which would have provided a more complete picture of drivers of CS in the study hospitals. Furthermore, this study was only conducted in public hospitals, which does not offer much insight on practices in private facilities. This was due to difficulties in obtaining approval to access medical records in private facilities. It is noteworthy that CS rates tend to be higher in private facilities in Egypt [19], so we expect non-medically indicated CS to be an even greater problem in private hospitals.

## Conclusion

Over-medicalization of the birth process in Egypt as manifest in overuse of caesarean delivery constitutes a critical public health issue that merits immediate action due to the unnecessary strain CS places on the health system. Our study presented an in-depth analysis of clinical and non-clinical factors that are associated with CS delivery. Evidence-based interventions that address these factors, such as disseminating delivery protocols and practice guidelines, training providers in VBAC, and strengthening hospitals’ monitoring and supervision systems are key to reducing this potentially avoidable surgical procedure which carries distinct risks to mothers and newborns [43, 44, 46].

## Data Availability

Data supporting the results of this paper can be found in the Population Council database and can be shared on reasonable request.

## Abbreviations

CS: Caesarean section
EDHS: Egypt Demographic and Health Survey
MoHP: Ministry of Health and Population
VBAC: Vaginal birth after caesarean

## Footnotes

1 Multiple answers were allowed

2 Multiple answers were allowed.

3 University and teaching hospitals are tertiary care facilities

## References

[1] Betrán AP, Ye J, Moller A-B, Zhang J, Gülmezoglu AM, Torloni MR (2016). The increasing trend in caesarean section rates: global, regional and National Estimates: 1990-2014. PLoS One.

[2] Ministry of Health and Population E-Z, and ICF International (2015). Egypt demographic and health survey 2014.

[3] Al Rifai RH (2017). Trend of caesarean deliveries in Egypt and its associated factors: evidence from national surveys. BMC pregnancy and childbirth.

[4] Betran AP, Torloni MR, Zhang JJ, Gülmezoglu AM, Section WHOWGoC (2016). WHO statement on caesarean section rates. BJOG.

[5] Ye J, Zhang J, Mikolajczyk R, Torloni MR, Gulmezoglu AM, Betran AP (2016). Association between rates of caesarean section and maternal and neonatal mortality in the 21st century: a worldwide population-based ecological study with longitudinal data. BJOG.

[6] Betran AP, Torloni MR, Zhang J, Ye J, Mikolajczyk R, Deneux-Tharaux C (2015). What is the optimal rate of caesarean section at population level?A systematic review of ecologic studies. Reprod Health.

[7] Festin MR, Laopaiboon M, Pattanittum P, Ewens MR, Henderson-Smart DJ, Crowther CA (2009). Caesarean section in four south east Asian countries: reasons for, rates, associated care practices and health outcomes. BMC Pregnancy Childbirth.

[8] Begum T, Rahman A, Nababan H, Hoque DME, Khan AF, Ali T (2017). Indications and determinants of caesarean section delivery: evidence from a population-based study in Matlab. Bangladesh PloS one.

[9] Gibbons L, Belizán J, A Lauer J, Betrán A, Merialdi M, Althabe F (2010). The Global Numbers and Costs of Additionally Needed and Unnecessary Caesarean Sections Performed per Year: Overuse as a Barrier to Universal Coverage Health Systems Financing.

[10] Mylonas I, Friese K (2015). Indications for and risks of elective cesarean section. Dtsch Arztebl Int.

[11] Aminu M, Utz B, Halim A, van den Broek N (2014). Reasons for performing a caesarean section in public hospitals in rural Bangladesh. BMC Pregnancy Childbirth.

[12] Oner C, Catak B, Sütlü S, Kilinç S (2016). Effect of social factors on cesarean birth in Primiparous women: a cross sectional study (social factors and cesarean birth). Iran J Public Health.

[13] Feng XL, Xu L, Guo Y, Ronsmans C (2012). Factors influencing rising caesarean section rates in China between 1988 and 2008. Bull World Health Organ.

[14] Poma PA (1999). Effects of obstetrician characteristics on cesarean delivery rates. A community hospital experience. Am J Obstet Gynecol.

[15] Goyert GL, Bottoms SF, Treadwell MC, Nehra PC (1989). The physician factor in cesarean birth rates. N Engl J Med.

[16] DeMott RK, Sandmire HF (1990). The Green Bay cesarean section study. I. the physician factor as a determinant of cesarean birth rates. Am J Obstet Gynecol.

[17] World Health Organization, United Nations Children’s Fund, United Nations Population Fund, World Bank, United Nations Population Division (2014). Trends in maternal mortality: 1990 to 2013.

[18] El-Zanaty F, Way A (2009). Egypt demographic and health survey 2008.

[19] Ministry of H, Population/Egypt, El Z, Associates/Egypt, International ICF (2015). Egypt Demographic and Health Survey 2014.

[20] Salem BZ, تاريخ وتطور الرعاية الصحية الأولية في مصر (2018). [History and evolution of primary health care in Egypt]: ATLAS PUBLISHING HOUSE.

[21] Ministry of Health and Population (2017). Operation Manual for Primary Healthcare 2016.

[22] Central Agency for Public Mobilization and Statistics. Number of live births.https://www.capmas.gov.eg/Pages/IndicatorsPage.aspx?page_id=6135&ind_id=1097. Accessed 2 February 2019.

[23] Kingdon C, Downe S, Betran AP (2018). Non-clinical interventions to reduce unnecessary caesarean section targeted at organisations, facilities and systems: systematic review of qualitative studies. PLoS One.

[24] Kyu HH, Shannon HS, Georgiades K, Boyle MH (2013). Caesarean delivery and neonatal mortality rates in 46 low- and middle-income countries: a propensity-score matching and meta-analysis of demographic and health survey data. Int J Epidemiol.

[25] MacDorman MF, Menacker F, Declercq E (2008). Cesarean birth in the United States: epidemiology, trends, and outcomes. Clin Perinatol.

[26] Menacker F, Declercq E, Macdorman MF (2006). Cesarean delivery: background, trends, and epidemiology. Semin Perinatol.

[27] Osterman MJ, Martin JA, Menacker F (2009). Expanded health data from the new birth certificate, 2006. Natl Vital Stat Rep.

[28] Denk CE, Kruse LK, Jain NJ (2006). Surveillance of cesarean section deliveries, New Jersey, 1999–2004. Birth (Berkeley, Calif).

[29] Barber EL, Lundsberg LS, Belanger K, Pettker CM, Funai EF, Illuzzi JL (2011). Indications contributing to the increasing cesarean delivery rate. Obstet Gynecol.

[30] Abdel-Aleem H, Amin AF, Shokry M, Radwan RA (2005). Therapeutic amnioinfusion for intrapartum fetal distress using a pediatric feeding tube. Int J Gynaecol Obstet.

[31] ACOG G (2010). ACOG Practice bulletin no. 115: Vaginal birth after previous cesarean delivery. Obstet Gynecol.

[32] Stamilio DM, DeFranco E, Pare E, Odibo AO, Peipert JF, Allsworth JE (2007). Short interpregnancy interval: risk of uterine rupture and complications of vaginal birth after cesarean delivery. Obstet Gynecol.

[33] Flamm BL, Goings JR, Liu Y, Wolde-Tsadik G (1994). Elective repeat cesarean delivery versus trial of labor: a prospective multicenter study. Obstet Gynecol.

[34] Guise JM, Denman MA, Emeis C, Marshall N, Walker M, Fu R (2010). Vaginal birth after cesarean: new insights on maternal and neonatal outcomes. Obstet Gynecol.

[35] Birth After Previous Caesarean Birth (2015). RCOG Green-top Guideline No 45.

[36] Vlachos G, Tsikouras P, Manav B, Trypsianis G, Liberis V, Karpathios S (2015). The effect of the use of a new type of partogram on the cesarean section rates. J Turk Ger Gynecol Assoc.

[37] Mathai M (2009). The partograph for the prevention of obstructed labor. Clin Obstet Gynecol.

[38] Brost BC, Goldenberg RL, Mercer BM, Iams JD, Meis PJ, Moawad AH (1997). The preterm prediction study: association of cesarean delivery with increases in maternal weight and body mass index. Am J Obstet Gynecol.

[39] Patel RR (2005). Team tAS, Peters TJ, team tAS, murphy DJ, team tAS. Prenatal risk factors for caesarean section. Analyses of the ALSPAC cohort of 12 944 women in England. Int J Epidemiol.

[40] Caughey AB, Sundaram V, Kaimal AJ, Gienger A, Cheng YW, McDonald KM (2009). Systematic review: elective induction of labor versus expectant management of pregnancy. Ann Intern Med.

[41] Wood S, Cooper S, Ross S (2014). Does induction of labour increase the risk of caesarean section? A systematic review and meta-analysis of trials in women with intact membranes. BJOG.

[42] Mishanina E, Rogozinska E, Thatthi T, Uddin-Khan R, Khan KS, Meads C (2014). Use of labour induction and risk of cesarean delivery: a systematic review and meta-analysis. CMAJ.

[43] Khunpradit S, Tavender E, Lumbiganon P, Laopaiboon M, Wasiak J, Gruen RL. Non-clinical interventions for reducing unnecessary caesarean section. Cochrane Database Syst Rev. 2011;(6):CD005528. 10.1002/14651858.CD005528.pub2 21678348

[44] Chen I, Opiyo N, Tavender E, Mortazhejri S, Rader T, Petkovic J, et al. Non-clinical interventions for reducing unnecessary caesarean section. Cochrane Database Syst Rev. 2018;9:CD005528. 10.1002/14651858.CD005528.pub3 PMC6513634 30264405

[45] Visser GHA, Ayres-de-Campos D, Barnea ER, de Bernis L, Di Renzo GC, Vidarte MFE (2018). FIGO position paper: how to stop the caesarean section epidemic. Lancet.

[46] Gardner K, Henry A, Thou S, Davis G, Miller T (2014). Improving VBAC rates: the combined impact of two management strategies. Aust N Z J Obstet Gynaecol.

